# Evaluating Multipollutant Exposure and Urban Air Quality: Pollutant Interrelationships, Neighborhood Variability, and Nitrogen Dioxide as a Proxy Pollutant

**DOI:** 10.1289/ehp.1306518

**Published:** 2013-11-13

**Authors:** Ilan Levy, Cristian Mihele, Gang Lu, Julie Narayan, Jeffrey R. Brook

**Affiliations:** Air Quality Processes Research Section, Environment Canada, Downsview, Ontario, Canada

## Abstract

Background: Although urban air pollution is a complex mix containing multiple constituents, studies of the health effects of long-term exposure often focus on a single pollutant as a proxy for the entire mixture. A better understanding of the component pollutant concentrations and interrelationships would be useful in epidemiological studies that exploit spatial differences in exposure by clarifying the extent to which measures of individual pollutants, particularly nitrogen dioxide (NO_2_), represent spatial patterns in the multipollutant mixture.

Objectives: We examined air pollutant concentrations and interrelationships at the intraurban scale to obtain insight into the nature of the urban mixture of air pollutants.

Methods: Mobile measurements of 23 air pollutants were taken systematically at high resolution in Montreal, Quebec, Canada, over 34 days in the winter, summer, and autumn of 2009.

Results: We observed variability in pollution levels and in the statistical correlations between different pollutants according to season and neighborhood. Nitrogen oxide species (nitric oxide, NO_2_, nitrogen oxides, and total oxidized nitrogen species) had the highest overall spatial correlations with the suite of pollutants measured. Ultrafine particles and hydrocarbon-like organic aerosol concentration, a derived measure used as a specific indicator of traffic particles, also had very high correlations.

Conclusions: Our findings indicate that the multipollutant mix varies considerably throughout the city, both in time and in space, and thus, no single pollutant would be a perfect proxy measure for the entire mix under all circumstances. However, based on overall average spatial correlations with the suite of pollutants measured, nitrogen oxide species appeared to be the best available indicators of spatial variation in exposure to the outdoor urban air pollutant mixture.

Citation: Levy I, Mihele C, Lu G, Narayan J, Brook JR. 2014. Evaluating multipollutant exposure and urban air quality: pollutant interrelationships, neighborhood variability, and nitrogen dioxide as a proxy pollutant. Environ Health Perspect 122:65–72; http://dx.doi.org/10.1289/ehp.1306518

## Introduction

Long-term cohort studies that examine effects of air pollution on human health depend on accurate estimates of pollution levels and their variability for large populations. Cohort studies that focus on large geographical domains or examine between-city differences in pollution levels typically have used relatively few central site measurements per city to characterize exposure contrasts (e.g., Dockery et al. 1993). In recent years, with an appreciation for within-city contrasts, there has been interest in the intraurban scale. For such small scales central site monitors are inadequate for characterizing the full breath of exposure variations.

Several approaches have been taken to resolve the spatial variability in the intraurban scales to assign exposures (Health Effects Institute 2010), such as road proximity, land use regression (LUR), three-dimensional air quality models, dispersion models, and hybrid modeling approaches (e.g., Sampson et al. 2011). These solutions are limited in that they depend on the accuracy and availability of input data (such as information on road networks, land use data, reported emissions, and meteorology) and the empirically-based models (e.g., LUR) typically predict the spatial pattern for a limited number of easily measured pollutants [e.g., nitrogen dioxide (NO_2_), nitrogen oxides (NO_x_), black carbon (BC)] derived from a limited number of short measurement campaigns (typically 2 weeks) that may not account for the full variability within a season or a year. Nonetheless, associations between these predicted single pollutant spatial contrasts (most often NO_2_) and health outcomes have been reported by a number of studies (e.g., Jerrett et al. 2009; Thiering et al. 2013).

The air we breathe holds a mix of pollutants, and the associations found in these health studies likely result from this mixture, and are not the sole effect of the proxy pollutant (Crouse et al. 2010). It is therefore necessary to examine the entire mix of pollutants (Dominici et al. 2010), addressing questions such as: How does the spatial pattern differ among pollutants? Do pollutants emitted from the same sources exhibit the same spatial patterns? Are statistical correlations between different pollutants constant over time? Are they constant over space? Such an evaluation of multipollutant patterns is important to better understand the magnitude of exposure assignment errors when using a single pollutant, the potential impact of such assignment errors on exposure–effect relationships and to ultimately understand the full effect of complex mixtures (Billionnet et al. 2012).

Measurements of multiple air pollutants were taken in the city of Montreal, Quebec, Canada, with a mobile laboratory at high spatiotemporal resolution over multiple days. This paper focuses on these outdoor air pollutant measurements and multipollutant spatial and seasonal contrasts with the aim of gaining a better understanding of multipollutant exposures in an urban environment. We examined multipollutant statistical correlations and seasonal variability in relationships among pollutants measured across Montreal, including NO_2_, carbonaceous particles, and ultrafine particles (UFP) as pollutants related to traffic emissions in urban areas and of considerable interest regarding potential health effects.

Our hypotheses are that seasonal and spatial variations exist, not only in the ambient levels of different pollutants but also in the correlations between them, and that pollutants emitted from similar sources are correlated spatially.

## Methods

**Figure 1:**
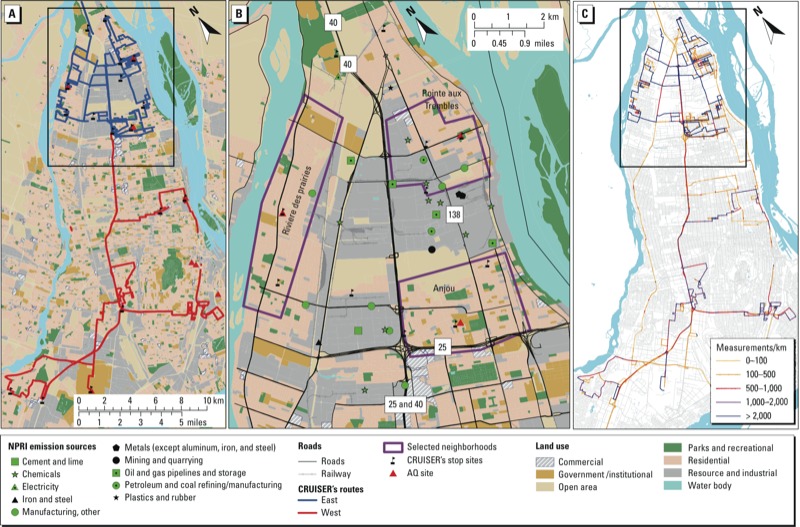
(*A*) Map of the study area showing land use types and CRUISER’s east (blue) and west (red) routes. (*B*) Higher resolution map showing the three neighborhoods of Anjou, Riviere des Prairies, and Point aux Tremble and major roads/highways, land use types, major emission sources, and CRUISER’s stop sites for the smaller area outlined in *A*. (*C*) The density of measurements per kilometer of road segment (measurements/km) based on all measurements combined.

The study area, measurements, sampling strategy and spatial analysis has been described previously (Levy et al. 2012). Briefly, mobile measurements of air quality and meteorological parameters were taken by Environment Canada’s mobile lab, the Canadian Regional and Urban Investigation System for Environmental Research (CRUISER), in 2009. The study was conducted on the Island of Montreal, which has a population of 1.8 million. As with many large cities, air pollution in Montreal is spatially variable. In addition to traffic, pollution sources on the island include oil and gas refining, storage, and distribution facilities, and oil and gas heaters (in the winter) (Environment Canada 2008). Maps of the study area that include information on land uses and the locations of main roads/highways and major point sources are shown in Figure 1.

The measurement campaign occurred during the winter (on 11 individual days, with the first on 13 January and the last on 11 February), summer (17 days between 8 July and 3 September), and autumn (6 days between 22 November and 2 December) of 2009. In addition to reporting results according to season, we report results for the 34 measurement days combined. Measurements were done on both weekdays (31 days) and weekends (3 days). Twenty-three pollutants (see Supplemental Material, Table S1) were measured simultaneously at time resolutions ranging from 0.5 sec to 2 min. Geo-location was recorded with a global positioning system at 1-sec intervals. All the measurements were organized according to 1-sec intervals by averaging finer time resolution measurements and repeating values every second for measurements with coarser resolutions.

Two driving routes were systematically followed: *a*) East Montreal, and *b*) Central and West Montreal. The east route was used most often (26 times, of which 11/11 were in the winter, 14/17 in the summer and 1/6 in the autumn) because of the greater number of industrial facilities, particularly petrochemical, operating in that part of the city. Focus in this area also provided additional data on exposures relevant to an asthma panel study conducted just after our measurements and that involved children residing in East Montreal (Dobbin et al. 2011). The impact of industry on exposure and respiratory health was one of the underlying objectives of both our mobile measurement campaign and the panel study. On each mobile measurement day the entire route (east or west) was completed to insure that all measurement locations (i.e., road segments) were visited on the same days. However, the time of day that each segment was visited was varied across the day to avoid bias due to typical diurnal variations. Analysis of the measurements was done by first assigning each 1-sec measurement to a road segment. For each road segment and each pollutant, we then calculated the daily average value for each measurement day, the seasonal average value over all days in the measurement season, and the overall average value for all measurement days. A road segment was usually a line connecting two junctions. For each pollutant, measurements from a given road segment were included in the analyses only if there were > 100 CRUISER measurements per kilometer of the segment among all measurement days, with measurements on at least 3 different days. For example, out of a maximum of 1,200 possible road segments (i.e., the number of segments CRUISER drove on at least once), 855 segments met these criteria for NO_2_ measurements, including 513 segments (60%) with measurements on ≥ 15 days, 624 (73%) that were 50–250 m in length, and 308 (36%) that were 50–100 m in length (the modal length for all segments).

To our knowledge, several of the pollutants included in this analysis (see Supplemental Material, Table S1) are unique to this study. NO_2_ is a specific measure of this species, as opposed to what is measured in regulatory air quality monitoring networks, which is typically biased by interferences from other oxidized forms of nitrogen [e.g., peroxyacetyl nitrate (PAN), nitric acid (HNO_3_), dinitrogen pentoxide (N_2_O_5_)] (Lee et al. 2011). Thus, NO_x_ is specifically nitric oxide (NO) + NO_2_ because of the more accurate measurement of NO_2_. The instrumentation used also provided a direct measurement of total oxidized nitrogen species (NO_y_), defined as NO_y_ = NO + NO_2_ + NO_z_. This latter class of compounds, NO_z_, represents the total quantity of nitrogen species that are more oxidized than NO_2_. NO_z_ is calculated from the direct measurements: NO_z_ = NO_y_ – NO_x_. Given their highly oxidized form (e.g., PAN, HNO_3_, N_2_O_5_) these “NO_z_ compounds” are of interest in terms of potential health effects (Brook et al. 2007). They are also a good indicator of photochemically processed urban air, which builds up during warm season stagnation (Luria et al. 2005). Also, as an additional indicator of oxidizing pollutants, we report O_x_, which is the sum of NO_2_ and ozone (O_3_). This measure remains constant when O_3_ is titrated by NO, a major reason for the observed spatial variability in O_3_ within cities. It is important to note that among the nitrogen-related compounds only NO and NO_y_ are measured directly, whereas NO_2_ is obtained by measuring NO_2_ + NO with one instrument and subtracting the NO measured by a different instrument. Measurements obtained through this approach (i.e., a difference technique), which includes NO_2_ and NO_z_ and those depending upon them (i.e., NO_x_ and NO_z_), have larger uncertainties compared with NO_y_ and NO.

We used an Aerodyne aerosol mass spectrometer (Aerodyne Research, Billerica, MA, USA) to provide 2-min resolution for the main contributors to PM_1_ (particulate matter ≤ 1 μm in vacuum aerodynamic diameter) mass; total organic matter (OM), sulfate (SO_4_), nitrate (NO_3_), ammonium (NH_4_), and independent mass fragment (*m/z*) measurements across the full mass spectrum (*m/z*). In this study, we focused on *m/z* = 57 because of its relationship to fuel combustion, and on hydrocarbon-like organic aerosol (HOA), a derived measure calculated from the *m/z* fragments by a source apportionment model that provides an estimate of the total mass of organic particles emitted from fossil fuel combustion (Levy et al. 2012). In cities, HOA is typically dominated by traffic exhaust.

Given that some emission sources vary by season (e.g., due to seasonal variation in heating or construction), and that some pollutants or their emissions are influenced by temperature, we hypothesized that there will be seasonal variation in ambient concentrations and multipollutant correlations, in addition to variation according to locations within the city. To test these hypotheses, we computed Pearson correlation coefficients (*r*_p_) between average levels of pollutants among all available road segments based on the year-round data, and separately for the winter, summer, and autumn seasons. Moreover, we calculated these spatial correlations separately for three different residential neighborhoods where measurements were conducted approximately the same number of times over the same days and seasons (locations are shown in Figure 1B). The Anjou neighborhood is in proximity to two major highways (Highways 25 and 40) and a major interchange; Riviere des Prairies (RdP), in the northwest part of the city, has much fewer industrial or traffic emission sources, but residents commonly use wood for residential heating (Gagnon et al. 2007); Point aux Tremble (PaT) is closer to the oil refineries and industrial emissions sources than the other two neighborhoods. Although correlations are presented for all pairs of the pollutants measured, we focused on NO_2_ and its relationship with BC and UFP because all three are related to combustion sources and are commonly used as indicators of traffic air pollutant exposure. We also compare NO_2_ with OM and HOA because these particle species can be specific to traffic (e.g., BC and UFP) and are unique to this study. In addition, we also calculated the average correlation of each individual pollutant with all other pollutants. Although this is not a standard metric, we report it as an indication of how well each pollutant performs as an overall indicator of spatiotemporal variability in the urban air pollutant mixture.

## Results

**Figure 2:**
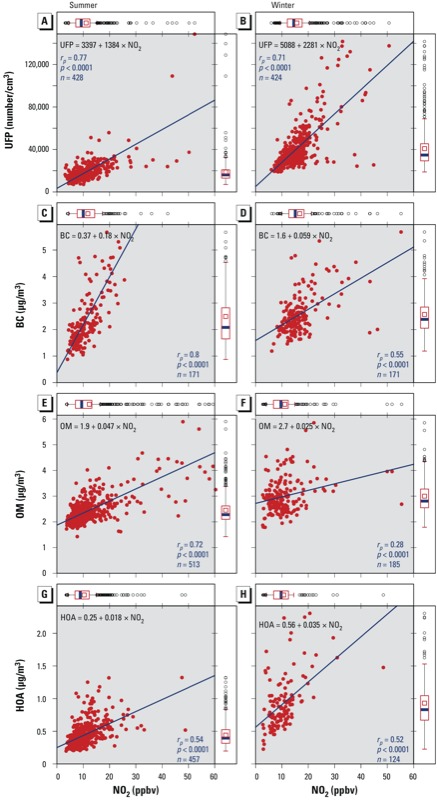
Scatter plots of UFP (*A,B*), BC (*C,D*), OM (*E,F*), and HOA (*G,H*) vs. NO_2_ (*x*-axes) for summer and winter measurements, with box plots on the top edge of each panel indicating the distribution of measurement data for NO_2_, and box plots on the right edge of each panel indicating the distribution of measurement data for the other pollutants. Each point in the scatter plots represents the average measurement at a road segment with ≥ 100 measurements/km on ≥ 3 days. Box plots indicate the mean (red square), median (blue line), high and low quartiles (outer red box), 1.5-IQR range (whiskers) and outliers (points). *r*_p_ is the Pearson’s correlation coefficient, *p* is the *p*-values of the correlation, and *n* is the sample size. The variation in *n* between seasons and pollutant pairs is due to varying numbers of measurement days and rates of data loss (i.e., due to quality assurance/quality control procedures and our criteria for data completeness for each segment). The equation at the top of each panel is the linear regression fit of the two pollutants, with the slope and intercept of each pair.

*Seasonal variation in the relationship between NO_2_ and particles*. Mean concentrations of all pollutants were higher during winter versus summer measurement days, with the exception of O_3_ and the volatile organic compounds (VOCs; benzene, C3 benzene, toluene, and xylenes) (data not shown). Figure 2 shows plots of mean values of NO_2_ versus mean values of UFP, BC, OM, and HOA measured at corresponding road segments during summer and winter measurement days. Focusing on UFPs, the winter median and mean (~ 36,000 and ~ 41,000, respectively) are double the corresponding values measured in the summer (~ 17,000 and ~ 19,000, respectively), consistent with a greater buildup due to reduced evaporation in the winter. Although Pearson correlation coefficients between UFPs and NO_2_ are similar for the winter and summer (0.71 and 0.77, respectively), a 1-ppbv increase in NO_2_ was associated with a larger increase in the number of UFPs during the winter than in the summer (2,281 UFPs/cm^3^ vs. 1,384 UFPs/cm^3^) (Figure 2A and 2B, respectively).

For BC, the correlation with NO_2_ was smaller in winter than summer (*r*_p_ = 0.55 vs. 0.80, respectively), along with higher NO_2_ mean and median in the winter (Figure 2C,D). In contrast with UFP, a 1-ppbv increase in NO_2_ was associated with a larger increase in BC during the summer than in the winter (0.181 vs. 0.059 μg/m^3^) (Figure 2C and 2D, respectively). The median concentration of OM was higher during the winter than the summer (~ 3 vs. ~ 2.2 μg/m^3^), but its correlation with NO_2_ was weaker in the winter (0.28 vs. 0.72) (Figure 2E,F). The median concentration of HOA also was larger in winter than in summer (0.82 vs. 0.40 μg/m^3^), but correlations with NO_2_ were similar for both seasons (*r*_p_~ 0.53) (Figure 2G,H). As for UFPs, a 1-ppbv increase in NO_2_ was associated with a larger increase in the HOA concentration during the winter than in the summer (0.035 vs. 0.018 μg/m^3^).

**Figure 3:**
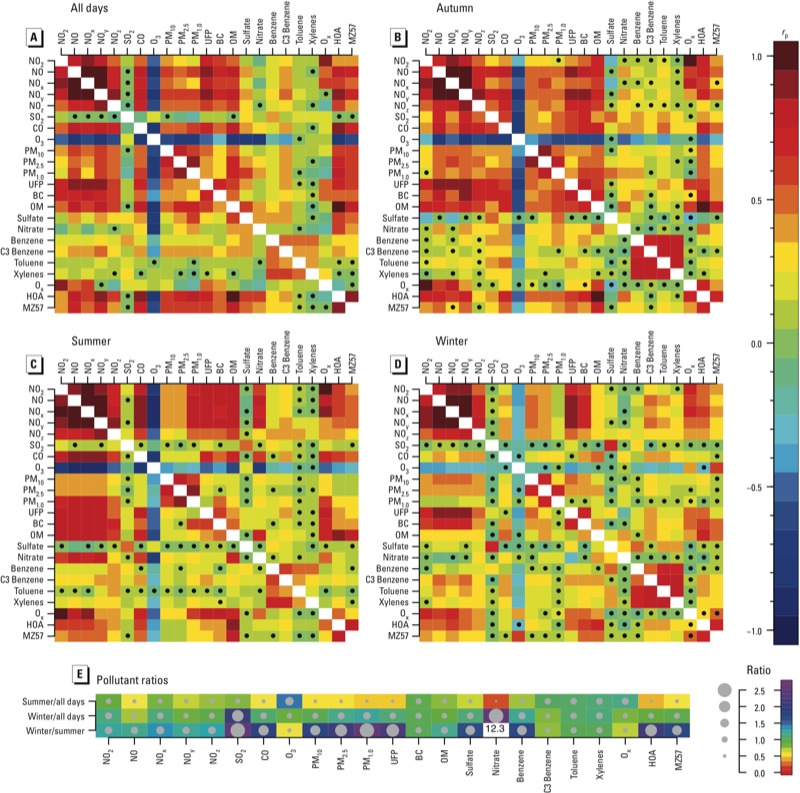
Pearson correlation coefficients for pairs of pollutants for all measurement days combined (*A*; 34 measurement days), and for measurement days in the autumn (*B*; 6 days), summer (*C*; 17 days), and winter (*D*; 11 days), with nonsignificant correlations (*p* > 0.05) indicated by a black dot, and the magnitude of each correlation indicated on the color bar to the right. Numeric data corresponding to *A–D* are provided in Supplemental Material, Tables S2–S5. (*E*) Ratios of mean pollutant levels measured in the summer and winter compared with mean values based on all measurement days combined, and ratios of mean pollutant levels measured in the winter compared with the summer.

*Multipollutant statistical correlations*. Seasonal variability in multipollutant correlations and concentrations. Correlation matrix plots in Figure 3 show relatively strong correlations among the nitrogen species or classes (NO_2_, NO, NO_x_, NO_y_, and NO_z_) based on combined data for all measurement days (*r*_p_ = 0.70–0.99, *p*-values < 0.001) (Figure 3A; see also Supplemental Material, Table S2).

In general, NO and NO_y_ were more strongly correlated with other pollutants than NO_2_. For example, the overall correlation coefficients with UFP were 0.89 for both NO_y_ and NO, compared with 0.63 and 0.80 for NO_2_ and NO_x_, respectively) (Figure 3A; see also Supplemental Material Table S2). Average correlations with all other pollutants combined based on all measurement days (see Supplemental Material, Table S2) suggest that NO_y_ and NO [average *r*_p_ (*r*_avg_) = 0.53] are slightly better overall indicators than NO_2_ and NO_x_ (*r*_avg_ = 0.40 and 0.48, respectively).

Other combustion-related pollutants, such as carbon monoxide (CO), O_3_, UFP, OM, and HOA also show good correlations with most pollutants (*r*_avg_ = 0.46–0.55), except for sulfur dioxide (SO_2_), and the VOCs (which also have important non-combustion sources) (Figure 3). Interestingly, HOA (i.e., traffic-related particles) had the highest average spatial correlation with the other pollutants (*r*_avg_ = 0.55 for all measurement days combined) (see Supplemental Material, Table S2). Of the measured gases, NO_y_ and CO had the strongest correlations with HOA (*r*_p_ = 0.82 and 0.84 for all days combined, respectively) (Figure 3).

For SO_2_ and the VOCs, there were almost no correlations with other species (e.g., *r*_p_ < 0.17 for SO_2_). This is not surprising given that the spatial patterns observed for these pollutants show highest concentrations near industrial emissions and not along roads (Levy et al. 2012). The VOCs are well correlated among themselves, particularly in the autumn and winter seasons (Figure 3B and 3D, respectively). For example, *r*_p_ between toluene and xylenes is 0.75 and 0.78 in the autumn and winter, respectively (see Supplemental Material, Tables S5 and S4, respectively). The different size fractions of particulate matter [PM_10_ (≤ 10 μm), PM_2.5_ (≤ 2.5 μm), and PM_1.0_ (≤ 1.0 μm)] show good correlations among themselves (*r*_p_ = 0.56–0.85, *p*-values < 0.0001) and lower correlations with the nitrogen oxides group (*r*_p_ = 0.29–0.59, *p*-values < 0.0001).

In general, correlation coefficients were higher for measurements in the summer compared with correlations for measurements over all days combined (Figure 3C and 3A, respectively; see also Supplemental Material, Table S3), especially for the nitrogen species and CO, O_3_, PM_1.0_, UFP, BC, OM, NO_3_, and O_x_ (i.e., O_3_ + NO_2_). These correlations, however, decrease in the winter (Figure 3D). For example, the correlation between CO and NO was *r*_p_ = 0.79 for all days combined, 0.86 for summer measurement days, and 0.34 for winter measurement days. Correlations of PM_10_ and PM_2.5_ with other pollutants were weaker in the summer and winter than for all days combined, whereas correlations of PM_1.0_ with other pollutants were highest in the summer and lowest in the winter.

Apart from the correlations between different pollutants, the mix of air pollutants in each season is also influenced by the mean concentration of each pollutant. The ratios between the mean concentrations among all road segments included for different seasons are shown in Figure 3E. Higher mean concentrations were found in the winter compared with the summer for almost all pollutants, with the exception of O_3_, O_x_, and VOCs (Figure 3E). However, the magnitude of the winter increase varied among pollutants, further indicating that the characteristics of the mixture are not stable between seasons.

**Figure 4:**
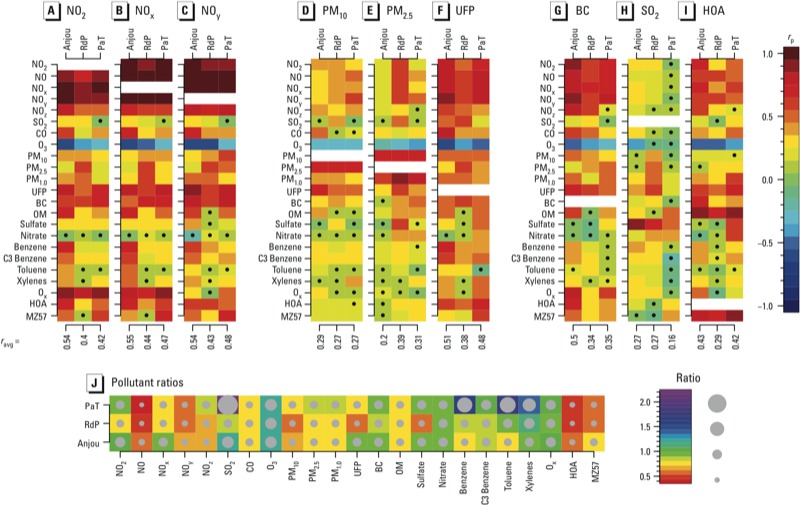
Pearson correlation coefficients between pairs of pollutants according to neighborhood [Anjou, Riviere des Prairies (RdP), and Point aux Tremble (PaT)] for selected pollutants [NO_2_ (*A*), NO_x_ (*B*), NO_Y_ (*C*), PM_10_ (*D*), PM_2.5_ (*E*), UFP (*F*), BC (*G*), SO_2_ (*H*), and HOA (*I*)] and mean absolute values of correlations between the selected pollutants and all other pollutants measured (*r*_avg_) according to neighborhood for all measurement days combined. (*J*) Ratios of the average correlations for each pollutant with all other pollutants in each neighborhood to the average correlation for the same pollutant with all other pollutant over the entire study area. All data are based on all measurement days combined. Nonsignificant correlations (*p* > 0.05) are indicated by a black dot, and the magnitude of each correlation or ratio is indicated on the color bar to the right.

Between-neighborhood variability in multipollutant correlations and concentrations. Figure 4A–I provide correlation maps of multipollutants for each neighborhood (same as Figure 3) focusing on nine pollutants, NO_2_, NO_x_, NO_y_, PM_10_, PM_2.5_, UFP, BC, SO_2_ and HOA. Supplemental Material Figure S1A–C shows the correlations for all pollutants. The ratios given in Figure 4J indicate which neighborhoods have higher pollution levels and the extent to which pollutants vary differently among neighborhood.

Although the correlations between nitrogen oxide species were high for all three neighborhoods (Figure 4A–C; see also Supplemental Material, Figure S1A–C), for each pollutant there were differences in the ratios of the average level in each neighborhood relative to the average for the study area as a whole (Figure 4J). For example, although the correlation between NO_x_ and NO in all three neighborhoods was about 0.97, the ratios of the average NO concentrations in each neighborhood relative to the entire study area were 0.64, 0.50, and 0.48 for Anjou, RdP, and PaT, respectively, suggesting large spatial variation within the city and considerably lower levels in the three neighborhoods than in other parts of the study areas. Average values of NO_x_ also were lower in the neighborhoods than in the study area as a whole; however, the differences were less pronounced (ratios of 0.96, 0.75, and 0.68, respectively), suggesting less spatial variation in NO_2_ between neighborhoods and supporting neighborhood-specific variation in the NO and NO_2_ mixture.

In general, across all three neighborhoods, average correlations of NO_2_, NO_x_, and NO_y_ with all other pollutants (0.40–0.54, 0.44–0.55, and 0.43–0.54, respectively) were higher than average correlations of PM_10_, PM_2.5_, UFP, BC, SO_2_, or HOA with all other pollutants (Figure 4A–I). Correlations for NO were also in these ranges. Similarly, UFPs also had relatively high average correlations (0.38–0.51), whereas PM_2.5_ and SO_2_ had low average correlations. Average correlations for HOA with all other pollutants were lower when calculated by neighborhood (correlations of 0.29–0.43) than when based on all available data (0.55; see Supplemental Material, Table S2), which included more measurements from main roads. This further suggests that multipollutant relationships differ spatially, with the nature of the sources playing a large role. In addition, average correlations for the combustion-related pollutants, (nitrogen species, UFP, BC, and HOA), which in most areas are strongly linked to traffic, were highest in the high-traffic, highway-influenced Anjou neighborhood (average values 0.43–0.55). In contrast with combustion-related pollutants, PM_2.5_ had a higher average correlation with other pollutants in the low-traffic, less industrially influenced Riviere des Prairies neighborhood than in the Anjou neighborhood (0.39 vs. 0.20). Lower average correlations with other pollutants for PM_2.5_ than other pollutants in all locations may at least partly reflect the low spatial variation of PM_2.5_ over the study area relative to other pollutants.

We previously observed that SO_2_, benzene, and toluene have the highest concentrations in proximity to the petrochemical emission sources (Levy et al. 2012). In the present study, we investigated the impact of the petrochemical industry and the use of SO_2_ as an indicator of these emissions in a quantitative approach by examining the statistical correlations of SO_2_ and other pollutants at different locations. We observed large differences among areas of the city for SO_2_ (as well as for benzene and toluene), with mean SO_2_ levels in the PaT neighborhood 2.2 times higher than mean levels in the study area as a whole (Figure 4J). Figure 4H further isolates the correlation of SO_2_ with the other pollutants by neighborhood, demonstrating different exposure patterns in different parts of the city. Interestingly, in the area closest and most affected by the petrochemical industries (PaT), SO_2_ exhibits the poorest correlation with the other pollutants (*r*_avg_ = 0.16). In contrast, in Anjou, which is located in a different direction from the petrochemical industries with respect to the prevailing westerly winds and further away (Figure 1), the correlations increased (*r*_avg_ = 0.27).

## Discussion

Our results demonstrate that there can be large differences in the intraurban spatial distributions of pollutants. Among the pollutants commonly used as human exposure indicators for epidemiological studies, correlations with other pollutants were relatively high for NO, NO_2_, NO_x_, and UFP, whereas correlations with PM_2.5_ and SO_2_ were relatively low. Although all these nitrogen species or classes were measured with the same method (high time-resolution chemiluminesence), which may tend to enhance their intercorrelations relative to those with other pollutants, this cannot entirely explain these correlations. Although our findings suggest that NO_y_ and HOA also may be good indicators of the mixture we measured, they are not included in typical monitoring network data, and inexpensive techniques to measure them are currently not available. Our results also showed seasonal differences in the correlations and relationships between pollutants, along with differences in their average concentrations.

The main reason for the spatial differences in correlations among pollutants was found to be the difference in their emission sources. Some pollutants are more linked to roads and traffic emissions (NO, NO_2_, UFP, and HOA), others to industrial sources (SO_2_ and benzene), and others to smaller, localized activities (Levy et al. 2012). Even for pollutants associated with common sources, such as SO_2_ and benzene, which are released from study area refineries, we observed small-scale differences in spatial patterns that reflect differences in the volumes of emissions from different sources (Environment Canada 2008), differences in the specific sources of emissions within an industrial complex (i.e., location and height), and differences in dispersion due to differences in their reactivity and physical characteristics. Thus, depending upon distance from the source and concentration averaging time, measurements may imply that certain pollutants covary when they are not physically linked. Consequently, there is potential for their exposure patterns within the population to differ and thus cause exposure misclassification. Although SO_2_ has been used as in indicator of exposure to refinery emissions in previous studies (Smargiassi et al. 2009), our observations suggest that it may not be an accurate indicator of specific aspects of the refinery emissions that could be more directly responsible for an adverse health effect in the Montreal study area.

Examining the nature of the relationship among pollutants in the mixture, we focused on NO_2_ and other traffic-related pollutants. We found a larger number of UFPs for each part-per-billion-by-volume increase of NO_2_ in the winter compared with the summer. Thus, in areas where NO_2_ is higher, there are greater amounts of UFPs in winter than in summer. This may reflect a reduced evaporation of UFPs co-emitted with NO_x_ when temperatures are colder (Olivares et al. 2007). HOA concentrations, a measure of traffic-related particles, were also more strongly associated with NO_2_ concentrations in winter than in summer, possibly due to a similar dependence on temperature.

In contrast with UFPs, associations between BC concentrations and NO_2_ were stronger in the summer than in the winter. This suggests a source for NO_2_ that emits less BC and/or a source for BC that emits less NO_2_ in the winter. One emitter that can explain the former is natural gas heating, which, although not the main source for heat, is used in Montreal (Statistics Canada 2010). For the latter, the lower combustion temperatures of wood burning for residential heating is a possible reason for a higher BC/NO_x_ emission ratio in winter, particularly in East Montreal (Gagnon et al. 2007). The higher wintertime OM concentration along with a lower OM correlation with NO_2_ further suggests an additional source for particles in the winter that does not produce as much NO_x_.

Beyond seasonal variation in emissions, there are two main reasons for the seasonality in pollution levels and correlations. First is the stable vertical structure in the lower atmosphere in the winter (Bergeron and Strachan 2012). Second, lower temperatures and reduced solar radiation in winter result in less photochemical activity, causing, for example, slower conversion of NO to NO_2_ and NO_2_ to NO_z_ and, therefore, more buildup of primary pollutants. Conversely, the photochemical production of O_3_ is higher in the summer because of stronger solar radiation, whereas UFPs and at least some portion of the traffic particle mass (i.e., HOA) either evaporates faster or does not form or condense in as high abundance as vehicle exhaust cools when ambient temperatures are higher in summer.

Differences in the seasonal behavior of air pollutants result not only in different levels of exposure to individual pollutants during each season but also in different correlations between pollutants, which may also vary depending upon their sources, thus reducing the accuracy of individual pollutants as proxy measures of chronic ambient air pollution exposures in urban areas. When combined with seasonality in population behavior, consideration of the seasonal differences in multipollutant behavior presented here can help lead to more-informed assessments of exposure contrasts in epidemiological studies. For example, the implication of the different NO_2_–UFP slopes between summer and winter is that if the association found between NO_2_ and health outcomes is due to UFP in the air pollution mix, then NO_2_ should show a stronger effect in the winter. This could be observable, assuming people spend the same amount of time outdoors in both seasons and if the indoor–outdoor air exchange rates do not vary between seasons. Nevertheless, the conclusion to be drawn is that NO_2_ has a nonconstant relationship during the year with some of the suspected causative pollutants. These are additional sources for uncertainty in epidemiological analysis that need to be addressed in order to confidently identify the pollutants or sources that are more responsible for the observed associations.

Given the limited number of pollutants available at monitoring sites to inform exposures for most health-related studies, it is important to consider their representativeness, particularly for NO_2_. However, few studies have taken spatial measurements of multiple pollutants to be able to quantitatively examine the associations of NO_2_ and other pollutants. Our results imply that although no single pollutant will capture the urban-scale variability in chronic human exposures to the air pollution mix as a whole, to a subset of exposures (e.g., traffic-related pollutants), nitrogen species (NO, NO_2_, NO_x_, and NO_y_), and to a lesser extent UFP, may be considered reasonably accurate proxy measures. This helps explain why estimates of chronic exposures to traffic-related air pollution by LUR models for NO_2_ has been useful in epidemiological studies. The higher correlations of NO_x_ and NO_y_ to other pollutants also suggest that detailed spatial maps for these pollutants may be more advantageous than NO_2_ for health studies, especially if the focus is on traffic-related air pollutants.

One limitation of this study is the representativeness of our road segment averages to chronic exposure conditions given the limited number of days and exclusion of evenings and nights. To assess this limitation, our averages were compared to the actual 2009 annual averages reported by Levy at al. (2012). As expected, a small sample of visits could not perfectly match the annual average; however, at the available monitoring sites CRUISER’s averages were within 25% of the observed values for NO_x_, NO_2_, O_3_, and PM_2.5_ and within 40% for CO and 31% for SO_2_. The average ratio of CRUISER to the network NO_x_ average was 0.96, suggesting that our mobile measurements attained a reasonable amount of long-term representativeness, while also covering a large range of urban settings.

The number of road segments meeting our criteria for computation of an average concentration varied by season and pollutant. This could affect our comparisons of correlations and regression results in terms of the magnitude and significance of the differences shown. Therefore, we expect that the most robust multipollutant correlations were those based on all of the data combined. Although a large number of the seasonal correlations were found to be significant, we did not test for significant differences among corresponding estimates by season or neighborhood. Another potential limitation is the restriction of our measurements to roads, which may not represent pollution levels at locations where people spend their time.

## Conclusions

The multipollutant correlations presented here characterize, both spatially and seasonally, the potential extent of the variability in the mix of air pollutants in urban areas. Not only do average levels of individual pollutants change from season to season, but correlations between pairs of pollutants can also vary by season. Furthermore, spatial correlations vary across the city. Consequently, no single pollutant can serve as a perfect proxy for the air pollution mix. However, among the more easily measured and often readily available pollutants, the nitrogen species (NO, NO_2_, NO_x_) continue to be the best compromise as proxy measures of urban-scale variability in chronic exposures to complex urban air pollution mixtures. In cities, such pollutants are strongly linked to traffic emissions but are not solely due to this source.

Conveying the tremendous amount of information that can be obtained through mobile surveys and extracting useful insights represents a challenge. The present study and Levy et al. (2012) provide examples of approaches that may be used to meet this challenge in the context of understanding chronic exposure. Our findings help confirm the degree of multi-scale complexity in urban outdoor air pollutant levels and the likelihood of substantial variability in individual exposures. Clearly, attempts to relate health end points to specific sources or industries and their mixtures through epidemiological studies must take this variability into consideration when assigning exposures and interpreting results.

## Supplemental Material

(1.5 MB) PDF

## References

[r1] Bergeron O Strachan IB 2012 Wintertime radiation and energy budget along an urbanization gradient in Montreal, Canada. Int J Climatol 32 137 152; 10.1002/joc.2246

[r2] Billionnet C Sherrill D Annesi-Maesano I 2012 Estimating the health effects of exposure to multi-pollutant mixture. Ann Epidemiol 22 126 141; 10.1016/j.annepidem.2011.11.004 22226033

[r3] Brook JR, Burnett RT, Dann TF, Cakmak S, Goldberg MS, Fan X (2007). Further interpretation of the acute effect of nitrogen dioxide observed in Canadian time-series studies.. J Expos Sci Environ Epidemiol.

[r4] Crouse DL Goldberg MS Ross NA Chen H Labrèche F 2010 Postmenopausal breast cancer is associated with exposure to traffic-related air pollution in Montreal, Canada: a case–control study. Environ Health Perspect 118 1578 1583; 10.1289/ehp.1002221 20923746 PMC2974696

[r5] Dobbin NA, Wheeler AJ, Smargiassi A, Bartlett S, Liu L, Shutt R, et al. (2011). Lung inflammation among children with asthma exposed to industrial and traffic pollution [Abstract 936]. In: 23rd Annual Conference of the International Society for Environmental Epidemiology (ISEE), 13–16 September 2011, Barcelona, Spain.. http://ehp.niehs.nih.gov/isee/PDF/isee11Abstract00936.pdf.

[r6] Dockery DW Pope CA III Xu X Spengler JD Ware JH Fay ME 1993 An association between air pollution and mortality in six U.S. cities. N Engl J Med 329 1753 1759; 10.1056/NEJM199312093292401 8179653

[r7] Dominici F Peng RD Barr CD Bell ML 2010 Protecting human health from air pollution. Epidemiology 21 187 194; 10.1097/EDE.0b013e3181cc86e8 20160561 PMC3478072

[r8] Environment Canada. (2008). National Pollutant Release Inventory. Tracking Pollution in Canada.. http://www.ec.gc.ca/inrp-npri/.

[r9] Gagnon C, Bessette C, Boulet D, Garneau Y, Paquette P, Mallet R. (2007). Air Quality in Montréal. 2007 Annual Report, City of Montréal.. http://ville.montreal.qc.ca/pls/portal/docs/page/enviro_fr/media/documents/rsqa_2007_en.pdf.

[r10] Health Effects Institute. (2010). Traffic-Related Air Pollution: A Critical Review of the Literature on Emissions, Exposure, and Health Effects. Special Report 17.. http://pubs.healtheffects.org/view.php?id=334.

[r11] Jerrett M Finkelstein MM Brook JR Arain MA Kanaroglou P Stieb DM 2009 A cohort study of traffic-related air pollution and mortality in Toronto, Ontario, Canada. Environ Health Perspect 117 772 777; 10.1289/ehp.11533 19479020 PMC2685840

[r12] Lee CJ Brook JR Evans GJ Martin RV Mihele C 2011 Novel application of satellite and in-situ measurements to map surface-level NO_2_ in the Great Lakes region. Atmos Chem Phys 11 11761 11775; 10.5194/acp-11-11761-2011

[r13] Levy I Mihele C Lu G Narayan J Hilker N Brook JR 2012 Elucidating multipollutant exposure across a complex metropolitan area by systematic deployment of a mobile laboratory. Atmos Chem Phys Discuss 12 31585 31627; 10.5194/acpd-12-31585-2012

[r14] Luria M Tanner RL Valente RJ Bairai ST Koracin D Gertler AW 2005 Local and transported pollution over San Diego, California. Atmos Environ 39 6765 6776; 10.1016/j.atmosenv.2005.07.051

[r15] Olivares G Johansson C Ström J Hansson HC 2007 The role of ambient temperature for particle number concentrations in a street canyon. Atmos Environ 41 2145 2155; 10.1016/j.atmosenv.2006.10.068

[r16] Sampson PD Szpiro AA Sheppard L Lindström J Kaufman JD 2011 Pragmatic estimation of a spatio-temporal air quality model with irregular monitoring data. Atmos Environ 45 6593 6606; 10.1016/j.atmosenv.2011.04.073

[r17] Smargiassi A Kosatsky T Hicks J Plante C Armstrong B Villeneuve PJ 2009 Risk of asthmatic episodes in children exposed to sulfur dioxide stack emissions from a refinery point source in Montreal, Canada. Environ Health Perspect 117 653 659; 10.1289/ehp.0800010 19440507 PMC2679612

[r18] Statistics Canada. (2010). Households and the Environment: Energy Use, 2007. Catalog no. 11-526-S.. http://www.statcan.gc.ca/pub/11-526-s/11-526-s2010001-eng.htm.

[r19] Thiering E, Cyrys J, Kratzsch J, Meisinger C, Hoffmann B, Berdel D (2013). Long-term exposure to traffic-related air pollution and insulin resistance in children: results from the GINIplus and LISAplus birth cohorts.. Diabetologia.

