# Supplementary material for: Evaluating Multipollutant Exposure and Urban Air Quality: Pollutant Interrelationships, Neighborhood Variability, and Nitrogen Dioxide as a Proxy Pollutant
Source: Environ Health Perspect. 2013 Nov 13;122(1):65–72. doi: 10.1289/ehp.1306518 (PMC3888565; doi:10.1289/ehp.1306518)
Supplement: (1.5 MB) PDF [file ehp.1306518.s001.508.pdf]

Environmental Health Perspectives doi:10.1289/ehp.1306518

Supplemental Material

Evaluating Multipollutant Exposure and Urban Air Quality: Pollutant Interrelationships, Neighborhood Variability, and Nitrogen Dioxide as a Proxy Pollutant

Ilan Levy, Cristian Mihele, Gang Lu, Julie Narayan, and Jeffrey R. Brook

Table of Contents

Supplemental Material, Table S1. List of pollutants measured by CRUISER and the methods employed……………………………………………………………………………………2

Supplemental Material, Table S2. Multipollutant correlations during the entire study campaign……………………………………………………………………………………4

Supplemental Material, Table S3. Multipollutant correlations during the summer measurements…………………………………………………………………………..…..5

Supplemental Material, Table S4. Multipollutant correlations during the winter measurements……………………………………………………………………………………6

Supplemental Material, Table S5. Multipollutant correlations during the autumn measurements……………………………………………………………………………………7

Supplemental Material, Figure S1. Pearson correlation coefficients (r_p) between pairs of pollutants according to neighborhood………..…………………………………..……………8

Supplemental Material, Table S1. List of pollutants measured by CRUISER and the methods employed.

Parameter	Avl[a]. (%)	Instrument model	Response time	Detection limit	Units
NO	80	Thermo Scientific / TECO 42CTL	1 sec	0.4 ppbv	ppbv
NO_2	64	Thermo Scientific / TECO 42CTL with Photolytic converter	1 sec	0.8 ppbv	ppbv
NO_y	62	Thermo Scientific / TECO 42CTL with Photolytic converter & Mo converter	1 sec	0.4 ppbv	ppbv
NO_x	64	Calculated ($NO + NO_2$)	NA	NA	ppbv
NO_z	46	Calculated (NO_y - NO_x)	NA	NA	ppbv
SO_2	79	Thermo Scientific / TECO 43 TLE with a 5-µm pore size Teflon filter	10 sec	1 ppbv	ppbv
CO	78	Thermo Scientific / TECO 48 with a 5-µm pore size Teflon filter	10 sec	100 ppbv	ppbv
O_3	79	Thermo Scientific / TECO 49	20 sec	1 ppbv	ppbv
O_x	53	Calculated ($NO_2 + O_3$)	NA	NA	ppbv
PM_{10}	88	GRIMM Dust Monitor 1.100	6 sec	0.1 µg/m^3	µg/m^3
$PM_{2.5}$	88	GRIMM Dust Monitor 1.100	6 sec	0.1 µg/m^3	µg/m^3
$PM_{1.0}$	88	GRIMM Dust Monitor 1.100	6 sec	0.1 µg/m^3	µg/m^3
UFP (Ultrafine particles)	87	GRIMM CPC 5.403	1 sec	0.6 # /cc	# /cc
BC (Black carbon)	49	Droplet Measurement Technologies / Photo Acoustic	1 sec	< 3.3 µg/m^3	µg/m^3
OM (Organic matter)	73	Aerodyne Aerosol Mass Spectrometer	2 min	0.15 µg/m^3	µg/m^3
Sulfate	73	Aerodyne Aerosol Mass Spectrometer	2 min	0.04 µg/m^3	µg/m^3
Nitrate	73	Aerodyne Aerosol Mass Spectrometer	2 min	0.02 µg/m^3	µg/m^3
HOA (Hydrocarbon-like organic aerosols)	36	Aerodyne Aerosol Mass Spectrometer (PMF application)	2 min	0.15 µg/m^3	µg/m^3
MZ57 (mass to charge ratio of 57)	36	Aerodyne Aerosol Mass Spectrometer	2 min	0.01 µg/m^3	µg/m^3
Benzene	73	IONICON High Sensitivity PTR-MS	10 sec	20 pptv	pptv

Parameter	Avl[a]. (%)	Instrument model	Response time	Detection limit	Units
C3 Benzene	73	IONICON High Sensitivity PTR-MS	10 sec	20 pptv	pptv
Toluene	73	IONICON High Sensitivity PTR-MS	10 sec	20 pptv	pptv
Xylenes	73	IONICON High Sensitivity PTR-MS	10 sec	20 pptv	pptv

[a] Availability: percent of valid measurements from entire campaign.

Supplemental Material, Table S2. Multipollutant correlations during the entire study campaign.[a]

	r_{avg}	**NO**	**NO_x**	**NO_y**	**NO_z**	**SO_2**	**CO**	**O_3**	**PM_{10}**	**$PM_{2.5}$**	**$PM_{1.0}$**	**UFP**	**BC**	**OM**	**Sulfate**	**Nitrate**	**Benzene**	**C3 Benzene**	**Acetone**	**Toluene**	**Xylenes**	**O_x**	**HOA**	**MZ57**
NO_2	0.40	**0.78** (844)	**0.88** (844)	**0.74** (715)	**0.44** (716)	*0.11* (791)	**0.48** (784)	**-0.45** (775)	**0.39** (821)	**0.29** (821)	**0.22** (821)	**0.65** (730)	**0.71** (407)	**0.48** (753)	**0.12** (749)	-0.08 (753)	**0.17** (736)	**0.29** (730)	0.05 (740)	*0.10* (737)	**0.22** (725)	**0.85** (775)	**0.43** (593)	**0.40** (592)
NO	0.53		**0.98** (849)	**0.99** (998)	**0.71** (715)	-0.04 (1041)	**0.79** (1018)	**-0.74** (1018)	**0.56** (1053)	**0.50** (1053)	**0.53** (1053)	**0.88** (967)	**0.67** (583)	**0.75** (984)	**0.35** (982)	**0.11** (984)	**0.23** (975)	**0.42** (972)	0.03 (980)	*0.09* (976)	**0.12** (969)	**0.47** (766)	**0.80** (826)	**0.65** (826)
NO_x	0.48			**0.97** (716)	**0.69** (717)	0.04 (796)	**0.64** (786)	**-0.62** (776)	**0.48** (824)	**0.39** (824)	**0.33** (824)	**0.83** (733)	**0.73** (407)	**0.61** (757)	**0.17** (753)	-0.09 (757)	**0.22** (740)	**0.35** (734)	*0.10* (747)	*0.11* (742)	**0.23** (730)	**0.61** (775)	**0.60** (595)	**0.50** (594)
NO_y	0.53				**0.84** (716)	-0.03 (942)	**0.81** (930)	**-0.75** (893)	**0.59** (968)	**0.54** (968)	**0.59** (968)	**0.89** (902)	**0.67** (471)	**0.76** (853)	**0.39** (852)	**0.13** (853)	**0.27** (898)	**0.47** (893)	0.02 (904)	**0.16** (903)	**0.13** (895)	-0.01 (580)	**0.82** (720)	**0.66** (718)
NO_z	0.43					-0.07 (645)	**0.65** (640)	**-0.62** (585)	**0.48** (692)	**0.48** (692)	**0.50** (692)	**0.57** (621)	**0.61** (288)	**0.62** (578)	**0.25** (576)	-0.03 (578)	**0.18** (605)	**0.30** (592)	0.10 (612)	0.10 (610)	0.06 (603)	**-0.17** (581)	**0.61** (457)	**0.43** (457)
SO	0.15						*-0.08* (1050)	**0.17** (1074)	-0.04 (1084)	*-0.10* (1084)	**-0.15** (1084)	0.08 (983)	0.10 (595)	-0.04 (1005)	**0.30** (1002)	**0.12** (1005)	**0.36** (1004)	**0.11** (1002)	-0.01 (1011)	**0.21** (1008)	**0.31** (999)	**0.19** (742)	-0.01 (848)	0.02 (846)
CO	0.48							**-0.73** (1020)	**0.51** (1048)	**0.48** (1048)	**0.61** (1048)	**0.66** (970)	**0.50** (587)	**0.80** (975)	**0.42** (972)	**0.32** (975)	**0.22** (970)	**0.41** (967)	-0.01 (976)	*0.09* (976)	0.01 (957)	**0.16** (698)	**0.84** (821)	**0.65** (820)
O_3	0.50								**-0.53** (1050)	**-0.64** (1050)	**-0.70** (1050)	**-0.50** (949)	**-0.38** (559)	**-0.80** (994)	**-0.64** (992)	**-0.75** (994)	**-0.17** (961)	**-0.35** (963)	-0.02 (965)	0.02 (965)	*0.09* (953)	0.09 (775)	**-0.79** (818)	**-0.61** (817)
PM_{10}	0.39									**0.79** (1128)	**0.56** (1127)	**0.51** (995)	**0.34** (647)	**0.51** (1031)	**0.22** (1031)	**0.12** (1031)	**0.24** (1029)	**0.39** (1024)	0.02 (1032)	**0.11** (1033)	**0.14** (1019)	**0.20** (739)	**0.61** (880)	**0.54** (878)
$PM_{2.5}$	0.41										**0.85** (1127)	**0.40** (995)	**0.34** (647)	**0.63** (1031)	**0.42** (1031)	**0.26** (1031)	**0.19** (1029)	**0.37** (1024)	0.03 (1032)	0.06 (1033)	0.05 (1019)	-0.08 (739)	**0.71** (880)	**0.58** (878)
$PM_{1.0}$	0.43											**0.38** (995)	**0.33** (647)	**0.70** (1031)	**0.55** (1031)	**0.35** (1031)	**0.19** (1026)	**0.36** (1023)	0.01 (1030)	0.02 (1030)	-0.06 (1016)	**-0.18** (739)	**0.78** (880)	**0.57** (878)
UFP	0.46												**0.69** (645)	**0.61** (920)	**0.33** (918)	**0.13** (920)	**0.21** (964)	**0.36** (962)	-0.01 (1025)	0.06 (967)	0.06 (954)	**0.44** (637)	**0.68** (858)	**0.56** (857)
BC	0.42													**0.38** (489)	**0.19** (484)	0.10 (489)	**0.13** (579)	**0.27** (572)	0.00 (584)	*0.11* (582)	**0.15** (568)	**0.39** (309)	**0.51** (427)	**0.44** (424)
OM	0.51														**0.57** (1045)	**0.35** (1045)	**0.24** (936)	**0.37** (927)	-0.04 (943)	0.08 (940)	-0.00 (922)	*0.10* (690)	**0.97** (886)	**0.80** (884)
Sulfate	0.38															**0.40** (1045)	**0.42** (935)	**0.36** (925)	-0.02 (942)	**0.13** (939)	**0.12** (921)	-0.10 (686)	**0.62** (885)	**0.48** (883)
Nitrate	0.27																*0.10* (936)	*0.09* (927)	-0.03 (943)	-0.04 (940)	-0.09 (922)	**-0.22** (690)	**0.55** (886)	**0.42** (884)
Benzene	0.28																	**0.50** (1043)	0.04 (1053)	**0.50** (1054)	**0.56** (1036)	0.11 (627)	**0.31** (828)	**0.25** (827)
C3 Benzene	0.35																		**0.13** (1049)	**0.41** (1048)	**0.56** (1036)	**0.17** (632)	**0.39** (816)	**0.33** (815)
Acetone	0.09																			**0.11** (1058)	**0.21** (1044)	0.08 (636)	*-0.10* (837)	-0.07 (835)
Toluene	0.17																				**0.47** (1044)	*0.13* (634)	0.05 (831)	0.05 (831)
Xylenes	0.21																					**0.14** (633)	-0.07 (818)	-0.02 (818)
O_x	0.25																						**-0.20** (535)	-0.01 (533)
HOA	0.55																							**0.81** (920)

[a]Pearson's correlation coefficients. In parentheses: number of road segments meeting the criterions of more than 100 visits per km, more 100 visits in total and spread over 3 days or more. Bold font marks p-values ≤ 0.001, italic font marks p-values between 0.001 and 0.01.

Supplemental Material Table S3. Multipollutant correlations during the summer measurements.[a]

Summer	r_{avg}	NO	NO_x	NO_y	NO_z	SO_2	CO	O_3	PM_{10}	$PM_{2.5}$	$PM_{1.0}$	UFP	BC	OM	Sulfate	Nitrate	Benzene	C3 Benzene	Acetone	Toluene	Xylenes	O_x	HOA	MZ57
NO_2	0.52	**0.89** (503)	**0.94** (518)	**0.88** (277)	**0.70** (277)	**0.17** (477)	**0.77** (452)	**-0.74** (514)	**0.35** (505)	**0.34** (505)	**0.70** (505)	**0.77** (428)	**0.80** (171)	**0.72** (513)	0.01 (508)	**0.60** (513)	**0.17** (382)	**0.30** (385)	0.10 (391)	0.05 (387)	**0.22** (381)	**0.93** (514)	**0.54** (457)	**0.42** (454)
NO	0.55		**0.99** (504)	**0.99** (373)	**0.67** (269)	0.08 (519)	**0.86** (509)	**-0.84** (556)	**0.38** (544)	**0.36** (544)	**0.76** (544)	**0.81** (466)	**0.80** (194)	**0.76** (550)	-0.09 (549)	**0.77** (550)	*0.15* (423)	**0.33** (425)	**0.26** (426)	0.06 (423)	**0.25** (417)	**0.75** (501)	**0.53** (491)	**0.44** (486)
NO_x	0.53			**0.97** (279)	**0.72** (279)	0.10 (477)	**0.86** (452)	**-0.82** (514)	**0.37** (505)	**0.34** (505)	**0.72** (505)	**0.80** (429)	**0.80** (172)	**0.76** (513)	-0.06 (508)	**0.71** (513)	*0.15* (383)	**0.30** (386)	*0.13* (391)	0.04 (387)	**0.19** (381)	**0.82** (514)	**0.55** (459)	**0.44** (456)
NO_y	0.55				**0.87** (279)	0.07 (340)	**0.87** (335)	**-0.85** (380)	**0.34** (369)	**0.35** (369)	**0.75** (369)	**0.80** (276)	**0.80** (89)	**0.80** (378)	-0.03 (367)	**0.80** (378)	**0.21** (247)	**0.37** (247)	0.10 (256)	0.16 (245)	**0.30** (244)	**0.39** (276)	**0.51** (320)	**0.43** (316)
NO_z	0.44					0.14 (244)	**0.60** (237)	**-0.51** (278)	**0.32** (273)	**0.23** (273)	**0.49** (273)	**0.51** (203)	**0.68** (58)	**0.53** (270)	0.05 (266)	**0.33** (270)	**0.38** (181)	**0.34** (183)	0.11 (186)	**0.36** (185)	**0.27** (183)	**0.36** (276)	**0.40** (236)	**0.29** (233)
SO_2	0.16						0.07 (519)	-0.09 (572)	0.08 (549)	0.03 (549)	0.05 (549)	*0.15* (471)	*0.18* (205)	**0.14** (562)	**0.48** (558)	0.07 (562)	**0.33** (440)	**0.18** (443)	0.09 (446)	0.09 (440)	*0.12* (437)	**0.17** (477)	*0.13* (494)	0.06 (491)
CO	0.44							**-0.74** (550)	**0.21** (535)	**0.15** (535)	**0.24** (535)	**0.73** (469)	**0.46** (200)	**0.62** (546)	-0.02 (543)	**0.58** (546)	0.09 (427)	**0.35** (427)	**0.18** (438)	0.06 (433)	**0.17** (424)	**0.63** (452)	**0.55** (491)	**0.45** (489)
O_3	0.44								**-0.39** (572)	**-0.20** (572)	**-0.28** (572)	**-0.44** (511)	**-0.51** (229)	**-0.58** (590)	**0.17** (587)	**-0.71** (590)	*-0.15* (456)	**-0.24** (458)	-0.07 (468)	-0.07 (460)	**-0.25** (456)	**-0.45** (514)	**-0.43** (531)	**-0.33** (529)
PM_{10}	0.28									**0.74** (574)	**0.59** (574)	**0.26** (501)	**0.17** (226)	**0.21** (571)	-0.03 (569)	**0.31** (571)	**0.16** (453)	**0.18** (459)	0.08 (467)	0.09 (459)	**0.20** (455)	**0.27** (501)	0.11 (523)	*0.10* (521)
$PM_{2.5}$	0.25										**0.87** (574)	*0.15* (501)	0.10 (226)	**0.19** (571)	0.01 (569)	**0.19** (571)	0.08 (453)	0.09 (459)	0.08 (467)	0.04 (459)	0.10 (455)	**0.28** (501)	0.10 (523)	*0.12* (521)
$PM_{1.0}$	0.40											*0.13* (501)	**0.55** (226)	**0.28** (571)	0.03 (569)	**0.32** (571)	**0.22** (453)	**0.31** (459)	**0.15** (467)	0.08 (459)	**0.28** (455)	**0.60** (501)	**0.32** (523)	**0.27** (521)
UFP	0.40												**0.54** (183)	**0.40** (509)	0.08 (504)	**0.17** (509)	**0.20** (386)	**0.38** (388)	-0.02 (434)	0.07 (396)	**0.22** (388)	**0.73** (426)	**0.38** (486)	**0.33** (481)
BC	0.43													**0.48** (230)	0.11 (226)	0.14 (230)	0.01 (165)	0.16 (167)	-0.04 (176)	0.03 (170)	0.15 (169)	**0.75** (171)	**0.37** (206)	**0.36** (204)
OM	0.47														0.02 (589)	**0.69** (593)	**0.21** (459)	**0.26** (461)	0.02 (471)	**0.20** (463)	*0.13* (458)	**0.64** (509)	**0.87** (533)	**0.72** (531)
Sulfate	0.17															0.01 (589)	**0.28** (457)	**0.21** (460)	0.06 (468)	0.14 (460)	**0.19** (455)	0.08 (504)	0.10 (531)	0.08 (529)
Nitrate	0.38																0.07 (459)	**0.18** (461)	0.04 (471)	0.07 (463)	0.11 (458)	**0.44** (509)	**0.17** (533)	*0.12* (531)
Benzene	0.25																	**0.57** (455)	0.07 (461)	**0.50** (458)	**0.64** (450)	0.13 (381)	**0.23** (446)	0.07 (446)
C3 Benzene	0.30																		*0.12* (462)	**0.38** (456)	**0.69** (452)	**0.22** (383)	**0.31** (449)	**0.19** (447)
Acetone	0.13																			0.07 (464)	**0.22** (459)	*0.14* (389)	0.05 (455)	0.00 (452)
Toluene	0.17																				**0.43** (452)	0.03 (386)	*0.14* (449)	0.02 (448)
Xylenes	0.27																					0.00 (387)	0.10 (451)	0.03 (449)
O_x	0.44																						**0.44** (454)	**0.32** (451)
HOA	0.38																							**0.72** (539)

[a]Pearson's correlation coefficients. In brackets: number of road segments meeting the criterions of more than 100 visits per km, more 100 visits in total and spread over 3 days or more. Bold font marks p-values ≤ 0.001, italic font marks p-values between 0.001 and 0.01.

Supplemental Material Table S4. Multipollutant correlations during the winter measurements.[a]

**Winter**	r_{avg}	**NO**	**NO_x**	**NO_y**	**NO_z**	**SO_2**	**CO**	**O_3**	**PM_{10}**	**$PM_{2.5}$**	**$PM_{1.0}$**	**UFP**	**BC**	**OM**	**Sulfate**	**Nitrate**	**Benzene**	**C3 Benzene**	**Acetone**	**Toluene**	**Xylenes**	**O_x**	**HOA**	**MZ57**
NO_2	0.37	**0.76** (423)	**0.88** (424)	**0.83** (362)	**0.48** (362)	0.04 (264)	*0.16* (295)	**-0.36** (176)	**0.35** (379)	**0.34** (379)	0.13 (379)	**0.71** (424)	**0.55** (171)	**0.28** (185)	0.09 (185)	0.01 (185)	0.04 (339)	**0.25** (322)	0.06 (341)	*0.15* (341)	0.10 (335)	**0.70** (176)	**0.52** (124)	**0.34** (124)
NO	0.45		**0.98** (425)	**0.98** (408)	**0.65** (362)	0.06 (310)	**0.34** (327)	**-0.34** (214)	**0.48** (418)	**0.39** (418)	0.10 (418)	**0.85** (461)	**0.71** (197)	**0.25** (219)	*0.18* (219)	-0.13 (219)	**0.21** (375)	**0.46** (364)	*0.17* (378)	**0.36** (379)	**0.29** (373)	**0.57** (175)	**0.52** (156)	**0.32** (156)
NO_x	0.44			**0.99** (363)	**0.64** (363)	0.05 (265)	**0.27** (295)	**-0.33** (177)	**0.46** (383)	**0.39** (383)	0.10 (383)	**0.83** (426)	**0.70** (172)	**0.28** (186)	0.15 (186)	-0.08 (186)	0.13 (340)	**0.37** (323)	*0.15* (342)	**0.29** (342)	**0.24** (337)	**0.63** (176)	**0.60** (125)	**0.36** (125)
NO_y	0.44				**0.72** (363)	0.04 (282)	**0.37** (300)	**-0.30** (160)	**0.46** (358)	**0.36** (358)	**0.19** (358)	**0.82** (411)	**0.65** (167)	*0.25* (166)	0.14 (166)	0.02 (166)	**0.20** (324)	**0.44** (309)	**0.20** (332)	**0.41** (332)	**0.33** (325)	**0.44** (132)	**0.45** (130)	**0.30** (130)
NO_z	0.38					-0.01 (238)	**0.35** (266)	*-0.24* (136)	**0.36** (327)	**0.37** (327)	**0.22** (327)	**0.48** (362)	0.36 (136)	**0.29** (147)	*0.23* (146)	*0.25* (147)	*0.18* (285)	**0.31** (273)	**0.25** (288)	**0.32** (287)	**0.28** (283)	**0.33** (135)	**0.40** (110)	**0.32** (110)
SO_2	0.13						-0.07 (257)	-0.16 (175)	-0.06 (267)	-0.05 (267)	-0.02 (267)	**0.23** (330)	-0.01 (124)	-0.08 (92)	**0.65** (92)	0.03 (92)	**0.26** (238)	-0.06 (225)	-0.15 (239)	0.09 (240)	0.09 (228)	0.00 (133)	-0.00 (77)	-0.04 (77)
CO	0.27							-0.15 (153)	**0.20** (318)	**0.30** (318)	**0.28** (318)	0.08 (347)	**0.40** (137)	**0.49** (145)	-0.07 (145)	*0.26* (145)	0.13 (276)	**0.42** (252)	**0.22** (277)	**0.39** (279)	**0.29** (274)	0.23 (113)	0.22 (124)	0.11 (124)
O_3	0.27								-0.16 (173)	-0.14 (174)	-0.02 (173)	**-0.38** (239)	-0.22 (80)	-0.09 (50)	-0.11 (50)	0.08 (50)	-0.20 (123)	**-0.31** (121)	-0.21 (122)	-0.22 (123)	**-0.30** (121)	**0.41** (176)	-0.30 (21)	*0.60* (21)
PM_{10}	0.28									**0.69** (429)	**0.31** (429)	**0.44** (428)	**0.33** (182)	0.16 (230)	0.04 (230)	-0.04 (230)	0.08 (380)	**0.26** (368)	0.11 (383)	*0.16* (384)	**0.18** (380)	*0.23* (132)	**0.27** (164)	0.13 (164)
$PM_{2.5}$	0.29										**0.63** (429)	**0.32** (428)	**0.42** (182)	**0.35** (230)	-0.05 (230)	**0.22** (230)	0.05 (380)	**0.26** (368)	*0.14* (383)	*0.15* (384)	*0.16* (380)	0.08 (133)	0.17 (164)	0.06 (164)
$PM_{1.0}$	0.18											0.08 (428)	0.08 (182)	*0.17* (230)	-0.04 (230)	*0.18* (230)	0.01 (380)	0.08 (368)	*0.17* (383)	0.09 (384)	0.07 (380)	0.16 (133)	0.04 (164)	0.05 (164)
UFP	0.39												**0.64** (214)	0.14 (233)	**0.26** (233)	**-0.25** (233)	**0.19** (388)	**0.36** (375)	-0.01 (392)	**0.22** (393)	**0.18** (386)	**0.49** (176)	**0.45** (165)	*0.21* (165)
BC	0.40													**0.41** (64)	-0.07 (64)	0.03 (64)	*0.23* (156)	**0.41** (143)	0.20 (158)	**0.32** (157)	**0.29** (150)	**0.47** (60)	**0.53** (36)	*0.51* (36)
OM	0.32														**0.21** (233)	**0.43** (233)	0.06 (210)	**0.36** (192)	0.09 (214)	**0.30** (210)	*0.21* (200)	0.36 (38)	**0.69** (165)	**0.52** (165)
Sulfate	0.24															**0.24** (233)	**0.47** (210)	0.07 (192)	0.02 (214)	**0.35** (210)	**0.34** (200)	-0.12 (38)	0.07 (165)	0.09 (165)
Nitrate	0.18																0.04 (210)	-0.02 (192)	0.01 (214)	0.04 (210)	0.06 (200)	-0.00 (38)	-0.08 (165)	0.11 (165)
Benzene	0.25																	**0.52** (372)	*0.13* (388)	**0.66** (388)	**0.68** (384)	-0.13 (95)	0.10 (148)	0.06 (148)
C3 Benzene	0.35																		**0.33** (375)	**0.76** (375)	**0.77** (370)	0.14 (94)	**0.39** (131)	**0.30** (131)
Acetone	0.19																			**0.41** (391)	**0.37** (384)	0.13 (95)	0.10 (151)	0.04 (151)
Toluene	0.34																				**0.78** (386)	-0.03 (95)	**0.37** (149)	**0.31** (149)
Xylenes	0.31																					-0.01 (95)	*0.26* (143)	0.18 (143)
O_x	0.31																						0.31 (17)	0.43 (17)
HOA	0.36																							**0.68** (184)

[a]Pearson's correlation coefficients. In brackets: number of road segments meeting the criterions of more than 100 visits per km, more 100 visits in total and spread over 3 days or more. Bold font marks p-values ≤ 0.001, italic font marks p-values between 0.001 and 0.01.

Supplemental Material Table S5. Multipollutant correlations during the autumn measurements.[a]

Autumn	r_{avg}	NO	NO_x	NO_y	NO_z	SO_2	CO	O_3	PM_{10}	$PM_{2.5}$	$PM_{1.0}$	UFP	BC	OM	Sulfate	Nitrate	Benzene	C3 Benzene	Acetone	Toluene	Xylenes	O_x	HOA	MZ57
NO_2	0.40	**0.68** (84)	**0.88** (84)	**0.74** (79)	**0.47** (79)	0.25 (81)	**0.40** (71)	*-0.33* (81)	0.30 (71)	0.26 (71)	0.17 (71)	**0.63** (81)	*0.52* (27)	**0.65** (56)	-0.26 (56)	0.07 (56)	0.12 (84)	0.14 (84)	*0.30* (84)	0.15 (84)	0.01 (83)	**0.97** (81)	**0.69** (44)	*0.39* (44)
NO	0.53		**0.96** (85)	**0.99** (301)	**0.70** (80)	**0.67** (305)	**0.73** (283)	**-0.70** (300)	**0.51** (295)	**0.44** (295)	**0.46** (295)	**0.89** (295)	**0.77** (152)	**0.66** (248)	0.01 (248)	**0.37** (248)	**0.22** (309)	0.12 (307)	**-0.24** (310)	**0.28** (310)	0.13 (303)	**0.59** (81)	**0.68** (225)	**0.47** (225)
NO_x	0.47			**0.97** (80)	**0.69** (80)	**0.34** (82)	**0.46** (72)	**-0.51** (82)	**0.51** (71)	**0.55** (71)	**0.41** (71)	**0.83** (82)	0.45 (28)	**0.55** (57)	-0.31 (57)	0.09 (57)	0.12 (84)	0.17 (85)	**0.37** (85)	0.23 (84)	0.07 (83)	**0.80** (81)	**0.54** (45)	0.29 (45)
NO_y	0.55				**0.85** (80)	**0.69** (302)	**0.76** (275)	**-0.73** (296)	**0.50** (291)	**0.47** (291)	**0.50** (291)	**0.89** (292)	**0.81** (149)	**0.69** (245)	0.00 (245)	**0.37** (245)	**0.26** (305)	0.14 (300)	**-0.20** (307)	**0.30** (307)	*0.19* (294)	**0.37** (77)	**0.69** (223)	**0.50** (223)
NO_z	0.43					**0.50** (79)	**0.62** (66)	**-0.59** (77)	**0.41** (70)	**0.46** (70)	*0.37* (70)	**0.71** (78)	**0.67** (26)	**0.61** (51)	0.11 (51)	0.29 (51)	0.15 (80)	0.18 (80)	0.25 (80)	0.18 (79)	0.11 (78)	0.07 (77)	0.31 (43)	0.13 (43)
SO_2	0.42						**0.59** (313)	**-0.63** (342)	**0.34** (332)	**0.29** (332)	**0.43** (332)	**0.65** (335)	**0.63** (175)	**0.38** (278)	**0.48** (278)	**0.30** (278)	**0.38** (348)	**0.29** (346)	*-0.15* (349)	**0.42** (348)	**0.27** (342)	0.11 (77)	**0.38** (259)	**0.28** (259)
CO	0.48							**-0.67** (307)	**0.35** (297)	**0.35** (297)	**0.49** (297)	**0.61** (307)	**0.63** (157)	**0.71** (258)	0.14 (258)	**0.39** (258)	**0.41** (312)	**0.31** (312)	-0.07 (316)	**0.49** (315)	**0.27** (308)	0.29 (68)	**0.71** (236)	**0.50** (236)
O_3	0.45								**-0.42** (324)	**-0.44** (324)	**-0.57** (324)	**-0.66** (327)	**-0.61** (170)	**-0.59** (267)	-0.08 (267)	**-0.46** (267)	**-0.33** (342)	-0.12 (340)	0.07 (344)	**-0.36** (343)	-0.13 (337)	-0.09 (81)	**-0.50** (246)	**-0.28** (246)
PM_{10}	0.37									**0.82** (338)	**0.49** (338)	**0.47** (322)	**0.39** (165)	**0.43** (274)	-0.00 (274)	**0.30** (274)	**0.23** (332)	*0.17* (329)	**-0.24** (336)	**0.26** (334)	0.12 (328)	0.22 (69)	**0.45** (255)	**0.28** (255)
$PM_{2.5}$	0.38										**0.79** (338)	**0.37** (322)	**0.46** (165)	**0.47** (274)	*0.16* (274)	**0.30** (274)	**0.26** (332)	0.12 (329)	**-0.22** (336)	**0.20** (334)	0.10 (328)	0.04 (69)	**0.43** (255)	**0.30** (255)
$PM_{1.0}$	0.41											**0.44** (322)	**0.54** (165)	**0.54** (274)	**0.44** (274)	**0.38** (274)	**0.35** (332)	0.08 (329)	**-0.34** (336)	**0.21** (334)	0.11 (328)	-0.07 (69)	**0.44** (255)	**0.35** (255)
UFP	0.51												**0.74** (173)	**0.56** (276)	0.03 (276)	**0.30** (276)	**0.26** (349)	**0.19** (347)	**-0.21** (350)	**0.36** (349)	**0.21** (345)	0.28 (78)	**0.58** (256)	**0.44** (256)
BC	0.48													**0.66** (132)	-0.01 (132)	*0.26* (132)	**0.34** (174)	0.14 (171)	-0.03 (176)	**0.27** (175)	0.15 (172)	0.32 (27)	**0.70** (106)	**0.42** (106)
OM	0.50														-0.07 (281)	**0.47** (281)	**0.22** (277)	0.02 (275)	**-0.26** (281)	*0.19* (280)	0.08 (268)	**0.55** (51)	**0.96** (261)	**0.68** (261)
Sulfate	0.21															*0.16* (281)	0.14 (277)	-0.01 (275)	**-0.24** (281)	-0.07 (280)	0.07 (268)	-0.25 (51)	-0.13 (261)	0.04 (261)
Nitrate	0.30																0.01 (277)	-0.08 (275)	-0.12 (281)	0.08 (280)	-0.02 (268)	0.02 (51)	**0.45** (261)	**0.29** (261)
Benzene	0.32																	**0.68** (351)	0.05 (355)	**0.73** (354)	**0.77** (350)	0.03 (81)	*0.19* (258)	**0.25** (258)
C3 Benzene	0.24																		**0.21** (354)	**0.78** (352)	**0.76** (344)	0.11 (81)	0.00 (256)	0.10 (256)
Acetone	0.22																			0.14 (355)	0.08 (352)	0.21 (81)	**-0.22** (261)	**-0.23** (261)
Toluene	0.33																				**0.75** (351)	0.04 (81)	*0.18* (260)	**0.24** (260)
Xylenes	0.24																					-0.04 (81)	0.08 (249)	*0.18* (249)
O_x	0.31																						**0.66** (38)	0.36 (38)
HOA	0.49																							**0.68** (280)

[a]Pearson's correlation coefficients. In brackets: number of road segments meeting the criterions of more than 100 visits per km, more 100 visits in total and spread over 3 days or more. Bold font marks p-values ≤ 0.001, italic font marks p-values between 0.001 and 0.01.

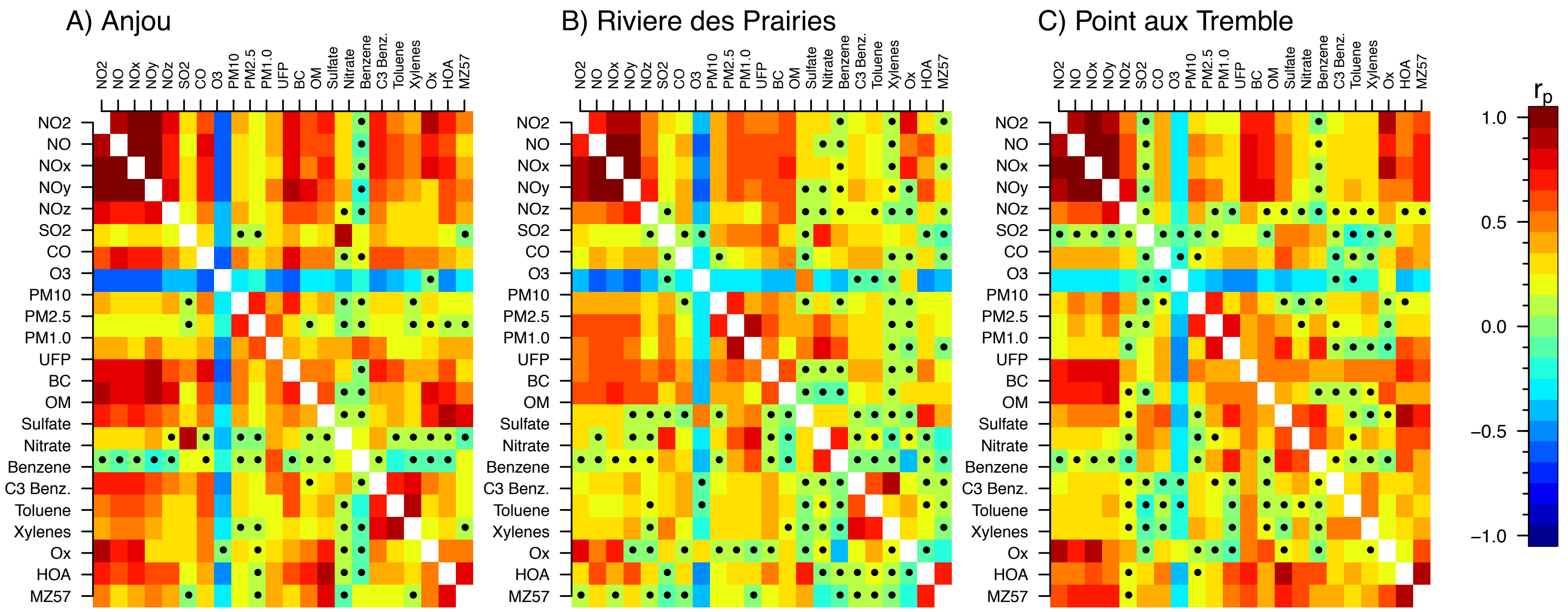

Supplemental Material, Figure S1. Pearson correlation coefficients (r_p) between pairs of pollutants according to neighborhood [(A) Anjou, (B) Riviere des Prairies (RdP), and (C) Point aux Tremble (PaT)]. All data are based on all measurement days combined. Non-significant correlations ($p > 0.05$) are indicated by a black dot, and the magnitude of each correlation is indicated on the color bar to the right.
